# Challenges in Implementing Integrated Care in Central and Eastern Europe – Experience of Poland

**DOI:** 10.5334/ijic.5533

**Published:** 2020-05-19

**Authors:** Donata Kurpas

**Affiliations:** 1 IFIC Board Member, IJIC Editorial Board Member, EURIPA, Wroclaw Medical University, PL

**Keywords:** Central and Eastern Europe, barriers, health care, patients’ needs, integrated care

## Abstract

During their transition, Central and Eastern European countries’ health and social care systems have undergone significant changes, and are currently dealing with serious problems of disintegration, coordination, and a lack of control over the market environment, especially for meeting patients’ needs. The increased health and social needs related to the ageing society and epidemiological patterns in these countries also require increased funding, reformation of rationing, sectors to be integrated (the managed care approach), and the development of an analytical information base for surveillance of new health and social care solutions.

## Context

The member states of the World Health Assembly have adopted a framework on integrated people-centered health services providing new direction and political commitment [1] to primary health care (PHC) as a crucial part of integrated care. On the fortieth anniversary of the World Health Organization’s (WHO) Alma-Ata Declaration, the Organization has reaffirmed the central role of PHC as part of its return to its basic tenets [2,3]. Primary health care’s strength varies greatly across Europe, with national and international policy initiatives granting it a range of differing responsibilities; the investment needed, e.g., to avoid future workforce shortages also varies [4].

The period of systems transitions in Central and Eastern European (CEE) countries entailed significant changes in their health systems, including health care financing. Large deficits in the public financing of health systems were just one of the challenges arising from the economic downturn of the 1990s, which in a number of CEE countries was coupled with inflation, increasing unemployment, low salaries, a large informal sector, and tax evasion. During the communist period there had been universal access to a wide range of health services, and it proved difficult to retain this coverage. Many states sought to ration publicly funded health services—for example, through patient cost-sharing or decreasing the scope of basic benefits. Yet not all these reform plans were implemented, and some were reversed due to the lack of social or political consensus. CEE health systems had also come to practice implicit rationing, in the form of under-the-table payments from patients, quasiformal payments to providers to compensate for a lack of funding, and long waiting lists forcing patients to the private sector. Patients in these countries are thus now confronted with a range of payment obligations for using health and social care services. Out-of-pocket payments now constitute a major source of health care financing [5,6].

## Patients’ needs in CEE countries

The authors of the report “Patient in the digital world How new technologies are changing the medical services market in Central and Eastern Europe” [7] concluded that CEE patients need access to information and are more and more aware of medical conditions. The internet and technology grant access to knowledge that was once available only to specialists. Patients are more frequently making use of the services they are entitled to. Patients on medical subscription services seek medical advice on average 3.6 times a year, up a fifth on the numbers from 2–3 years ago. This has a significant effect on the profitability of medical subscription and insurance. Patients want advice from a specialist—someone who they have selected themselves, who is well-known, and who is respected by other patients. The significance of doctor-rating systems is increasing. Small medical practices that offer individualized approach and continuity of care by the same doctor throughout the treatment are becoming an attractive alternative. Patients looking for a new doctor will regularly consider new opportunities and 30% of patients with a medical subscription or insurance purchase additional medical services. Yet only 35% actually visit the service provider for which they have bought the subscription or insurance; 65% instead select a totally different clinic or doctor. Patients are open to telemedical services and over 60% of patients in CEE state that they are interested in telemedical services. The important feature of this are the possibility of prompt advice and the ability to avoid queues. Patients are ready to use ‘health-related care’ and are ready not only to make use of specialized health care, but also medical services like dieticians and psychologists. This is the fastest growing segment in CEE region. Significantly, the cost is no longer the most important point. The range of services, the waiting time, and the proximity of the facility are also considered. Patients increasingly often book an appointment with a doctor by phone or on the internet, instead of by personal visit to the location, as was more common a few years ago. Patients are now much more open to using teleconsultations, which make getting an appointment quick and easy and allow access to a wider range of specialists. The preferred form is now video consultation, which show an increase from 10% to 25% of all teleconsultations over one year [7].

## Patients’ needs in Poland – OECD findings

### Current challenges

Poland’s record on mortality from treatable is relatively good among EU countries with similar or higher levels of expenditure on health, yet the mortality rate is still high and well above the EU average. Poland has historically relied heavily on inpatient care to provide health services. The excessive use of hospital care, as well as poor financial management, have resulted in a long-standing and unresolved problem of hospital indebtedness. Total health spending as a share of GDP (6.5%) and per capita (€1507) is among the lowest in the EU, as acknowledged by a recent pledge to increase public spending on health from 4.6% of GDP in recent years to 6.0% by 2024. At nearly 23% of health expenditure, out-of-pocket spending is fairly high. Most of this is related to the limited public coverage for outpatient pharmaceuticals. This has been the greatest single cause of catastrophic the health spending which affected about 30% of low-income households in 2014 [8].

At 2.4 per 1000 of population, the number of doctors is the lowest in the EU; this is also true of nurses, at 5.1 per 1000. The fraction of doctors who are general practitioners (GPs) is the second lowest in the EU, at 9% (the EU average is 23%). To alleviate this, pediatricians and internists are permitted to work as GPs. Medical personnel distribution varies widely: for example, the density of doctor varies by almost 70% from district to district. Some districts also suffer from a lack of certain medical specialists, including pediatricians, internists, anesthesiologists, and surgeons, in [8].

These weakness in outpatient care and shortages in health workforce lead to long waiting times, and go part way in explaining why certain indicators, such as unmet health care needs, are worse in Poland than in countries with similar levels of health spending. Average waiting times for specialist services was 3.4 months in 2018, with the longest times reported for endocrinology (11 months) and dentistry (8.5 months). Coordinated specialist post-heart-attack care was not contracted in seven districts in 2017 [8].

Health care governance is fragmented. This explains the slow progress of reforms aiming to reduce hospital beds and clear hospital debts. The highly fragmented nature of hospital ownership contributes to the lack of initiatives to reduce the number of beds [8].

### Goals for integrated care interventions

Life expectancy at birth has increased by 4 years in Poland since 2000, but is still three years less than the EU average. There are very significant inequalities in life expectancy by education and gender, with men with the lowest level of education living about 12 years shorted than the most educated. Life expectancy at age 65 has also increased, but two thirds of older people live with one or more chronic disease; almost half live with symptoms of depression. Statistics Poland projections suggest that there will be 10.8 million people aged 60 or over by 2030, and 13.7 million—40% of the population—by 2050 [8,9].

Unmet medical needs in Poland are higher than the EU average, and are mostly associated with long waiting times, though price is also a factor. One major coverage gap is medicine for outpatients, often leading to catastrophic spending in low-income households. There are issues Poland in access to (and quality of) PHC, which leads to the avoidable hospitalization rate for chronic conditions that could be treated in outpatient settings to be among the highest in Europe. Provision of care relies too much on hospitals; the shift to more community-based care has not yet occurred. Nonetheless, recent reforms and pilot schemes regarding coordination of care may assist here. The less than idea allocation of resources is reflected in hospital overcapacity, with the number of beds per head of population, already significantly higher than the EU average, remaining almost stable over the last ten years. However, a 2019 regulation ties nurse employment quotas to the number of beds, encouraging hospitals to reduce the number of beds, particularly given recent increases in nurses’ remuneration. The average hospital stay has decreased since 2005, now being below the EU average, and bed occupancy rate is now relatively low, too (at 66%, compared to 77% in the EU). All this suggests substantial room to release or reallocate hospital resources. Another area where efficiency can be potentially improved is the increased use of day surgery for certain procedures: day surgery for procedures cataract removal, inguinal hernia, and tonsillectomy are far below EU averages and (except for cataract removal) have remained unchanged since 2000. The main cause of this is medical procedures that require doctors to conservatively admit patients for observation [8].

A particular problem is Poland’s severe underdevelopment in formal long-term care and its subsequent heavy reliance on informal carers. Projections suggest that the number of dependent on others for the activities of daily living will increase by 30% over the next 50 years—faster than in the EU as a whole (25%) [8,10,11].

Behavioral risk factors account for almost half of all deaths. Both smoking rates and deaths from lung cancer have fallen, but remain are higher than the EU average; they are also much greater for men than for women. Obesity has increased over the last decade for adults, and especially in children; both remain below the EU average. Unhealthy dietary behavior and low physical activity also add to this growing health issue, to date broadly neglected [8]. As a fraction of current health expenditure, spending on disease prevention and health promotion is close to the EU average when measured, but per-person expenditure on preventive care is less than half the EU average (€34 compared to €89). The 2012–2015 national audit of preventive activities showed that financing of prevention was both lack and wrongly allocated [8,12]. Mortality from treatable causes remains much higher than the EU average; cancer survival rates are consistently lower, showing that there is great scope for improvements in early diagnosis and effective, timely treatment [8].

## Discussion

### Direction of integrated care initiatives in Poland

The 2017 legislative introduction of multidisciplinary PHC teams enhanced coordination of PHC by aligning care pathways, including posthospital rehabilitation and treatment. Coordination can also include activities in disease prevention and health promotion. This pilot approach will introduce elements of performance-linked payment in late 2020 [8].

### New competences of health care staff

The growth of the coordinated care focus has led to new responsibilities for Polish health professionals. Nurses and midwives with the appropriate training have since 2015 been able to prescribe some medicines and diagnostic tests. In the following year, ambulance personnel began working in hospital wards alongside nurses. The Ministry of Health proposed an increase in the number of medical secretaries in outpatient care in 2018, with the aim of helping to alleviate the administrative burden of doctors. The responsibilities of physiotherapists were broadened in 2019. Changes like these support more recent rules that require doctors to provide coordinated care for certain conditions, distributing tasks they would have traditionally performed to other health professionals. These expectations lack a formal definition, meaning that they may lead to initiatives that will allow skill mixes that change on the job. For instance, the legislation on coordinated oncological care fails to indicate who should take responsibility for coordination. The result in many hospitals has been that the coordinator role has been taken on by nurses, who have not received particular training for this [8].

A voluntary exchange in Poland in 2018 (hosted as part of the State of Health project in the EU cycle) discussed the notion of optimizing health workers’ roles as a means of addressing critical workforce shortages. Not as much attention has been paid to date improving integration between social care and health care. Social care is largely provided by family members, who receive very little support from the state. Despite this, a Sectorial Council on Competencies in Health and Social Care was established in 2016 and is currently exploring integration of this [8].

### Health technology

Maps of health needs have been available since 2015, but are not being used effectively in the support of decisions relating to investment and purchasing. The health technology assessment (HTA) agency has since 2015 appraised publicly funded health policy programs; a decision against a program can prevent health technologies deemed to be of low value from receiving funding. It may be that this approach can help improve the cost-effectiveness of the system’s resource allocation. Since 2016, a new system has assessed the costs and benefits of health care investments; thus is also expected to further improve the allocation of resources, including EU funds [8].

Further improvements in IT and eHealth solutions could contribute to improved efficiency [11]. All pharmacies and an increasing number providers of health care are currently connected to the e-prescription service, which will be mandatory from 2020. There is currently a pilot of an e-referrals system; this is expected to be deployed across the country in 2021. Mobile health solutions have not seen much adoption; what has been taken up is in the private sector, although social health insurance has covered prescriptions for mobile heart monitoring devices for a number of years [8].

### Hospital network

A 2017 ‘hospital network’ initiative helps integrate outpatient and inpatient care: participating hospitals receive a lump sum to set up specialist outpatient clinics covering both inpatient and outpatient care. Insurance contributions from an earmarked payroll tax funds most expenditure. Public funds covered about 70% of health funding in 2016, below the EU average of 79%. Out-of-pocket spending accounted for 23% of all health spending, with the bulk of it spent on outpatient medicines. About 6% of health expenditure goes on voluntary health insurance. Financial support from the European Structural and Investment Funds provides an important source of external funding for the system. In the 2014–20 financing period, Poland was allocated approximately EUR 2.8 billion for public health programs, improving quality of care, and developing eHealth solutions. Since 2016, new investments receiving public cofinancing (including EU funding) need to be formally evaluated to ensure cost-effectiveness and that they address local needs. Both these goals have recently been recognized by the EU: it has been underlined that that health care investment should account for regional disparities and that spending inefficiencies need addressing [8,13,14].

### Integrated care initiatives in primary health care

It has been suggested that quality initiatives could emphasize the skill mix, as doing so could help develop multidisciplinary PHC teams that are able to deliver complex personalized care while providing a better combination of curative and preventive services [8].

The National Health Fund (NHF), the public payer solely accountable for securing and organizing access to health care services in Poland, is responsible for implementing the Primary Health Care PLUS (PHC PLUS) project which aims to introduce a PHC centered model, based on coordinated, proactive and preventive methods relevant to patients’ needs and furthermore, works to keep patients well-informed and active participants in health care decision-making. Based on the agreement with the Ministry of Health dated November 28, 2017, the NHF currently facilitates the above project within an initiative entitled “Preparation, testing and implementation of Coordinated Care in the health care system. Stage 2, a pilot phase – Primary Health Care PLUS model”. Originally, the project was divided into three phases: 1. Creation of concept – developing 3 models; 2. Implementation of the model chosen for the pilot program; 3. Implementation of the final chosen measures throughout the entire health care system. Primary Health Care PLUS is composed of both regular PHC services and additional proactive and preventive activities based on plans provided by the PHC teams. It is focused more on preventive tools then on providing medical services after the fact. Objectives of the PHC PLUS include improving the quality of medical services at the PHC level, increasing the amount of medical services delivered at the PHC level instead of specialist and inpatient care, focusing on prevention rather than reaction, and coordination of medical services at the PHC level. All members of health professional teams should be regarded as patients’ partners, rather than just physicians. The level of supporting technology should be adequate to facilitate the overarching goals [15]. The available data (see – *Patients’ needs in Poland – OECD findings*) determined the scope of the medical interviews and physical examinations in periodic health examinations of adults as a part of this planned pilot study of coordinative care (the PHC PLUS project), the new, proactive, and complex model of Polish PHC. Periodic health examinations of adults were recommended for implementation in a model of coordinative care for PHC, which was suggested by the World Bank within the project ‘The preparation, testing and implementation in health care system of coordinated care organization (CCO)’ [16].

The implementation period of the project is July 1, 2018 through Dec. 31, 2021. Forty-two PHC PLUS providers attending to 288,392 patients are participating in the project. Approximately 1,100 medical staff members are involved in the project. PHC PLUS medical teams consist of specialists including physicians, coordinators, nurses, dietitians, psychologists, physiotherapists and health educators. Out of 41,022 health risk assessments declared to be conducted during the project, 18,058 (43.1%) were performed from July 1, 2018 to April 30, 2019, including 4,537 basic and 13,521 extended assessments. Furthermore, 15,020 patients in total, participated in the chronic disease management programs, which are also funded from the project. A coordinated care model in PHC in Poland can help train the focus on preventive interventions instead of on providing medical services to patients already diagnosed with diseases, many of which are already in more advanced stages. The primary health care population will be the main beneficiary of similar future programs. Other beneficiaries include the medical staff necessary for the proper implementation of integrated care models in CEE health care systems similar to the one that exists in Poland [15].

Future reform targets the entire population of Poland, with around 38 million people. This patient-oriented strategy seems to be better adapted to the current health care environment and demographic trends as, by the year 2060, the number of seniors in Poland is expected to double from 5.5 to 11 million and Poland will become one of the countries with the highest percentage of seniors in Europe [8,15,16].

## Recommendations for CEE countries on the basis of the Polish experience

Any implementation of an integrated care model that aims to be effective should incorporate human resource planning, in order to increase the number of PC professionals and address existing skill mix imbalances between specialists and GPs, as well as helping with the lack of personnel in nursing and allied health professions. A fully operational and interoperable e-communications system that is sensitive to the pragmatic conditions and which accommodates the needs of multidisciplinary teams. Effective use of ICT in a comprehensive medical records system should become a high priority for the health care service. Orienting the new PHC units to address major public health issues—such as cancer, CVD, diabetes, frailty, and traffic accidents—and risk factors—like smoking, obesity, driving behavior, and high consumption of sugar and alcohol—with interventions encompassing health promotion that aim to alter health care-seeking behaviors, prevention, screening and early diagnosis and management of health risks and disease as well as prevention (re)hospitalization. Coordinated actions for integrated chronic disease care and mobilizing resources beyond care structures, such as, through participatory initiatives on the level of community. In terms of policy, this involves substantial investment in information technology and training, strongly connecting the new approach offers with professional education and public awareness campaigns. Developing core competencies and implementing a coordinated continuing education program for PHC professionals: This focuses on the obvious need to retrain PHC practitioners in order to develop the PC team and nurture a culture of interdisciplinary collaboration. Interprofessional education through a national plan to restructure PHC training programs, with a focus on general practice training (structure, curriculum content, evaluation methods, and teaching) and other health science disciplines. Coordination of care by local and regional health authorities so as to link health care services with other domains and sectors that affect disease prevention and health promotion. Services should always be tailored to population health care needs, and policy planning should consider the public perception of PHC [3,17].

## Conclusions

People-centered coordinated care is expected to help care systems achieve the following objectives (Triple Aim goals): improving population health, increasing quality of care for the individual and lowering per capita costs [18]. However, we should remember that during the system transition in CEE countries, health care systems have undergone significant changes to adjust to the mainstream, leading to more autonomy for institutions and professions, the expansion of regional and local governance, and a greater role for market mechanisms. As a result, these states are now facing significant challenges with coordination, system disintegration, and a lack of control over the market environment (especially for medicines). Yet give the increased health needs of aging societies and epidemiological patterns, these countries need to increase funding, introduce rationing reforms, and improve cost-effectiveness, while integrating sectors through managed care and developing an information and analysis base to provide governance and supervision over uses of new technology [19].
